# Natural killer cell cryopreservation: Influence of composition, temperature, and granules on post-thaw survival and function

**DOI:** 10.1016/j.omta.2026.201813

**Published:** 2026-07-10

**Authors:** Adam Joules, Troy Louwagie, Akshat S. Mallya, Allison Hubel

**Affiliations:** 1 Department of Biomedical Engineering, University of Minnesota, Minneapolis, MN 55455, USA; 2 Department of Mechanical Engineering, University of Minnesota, Minneapolis, MN 55455, USA

**Keywords:** natural killer, cell therapy, cryopreservation, DMSO-free, differential evolution, Raman

## Abstract

Natural killer (NK) cells are promising off-the-shelf cancer immunotherapies, but their scalable deployment requires effective cryopreservation. Conventional dimethyl sulfoxide (DMSO)-based methods are associated with toxicity and persistent post-thaw functional impairment, and the mechanisms underlying NK cell cryoinjury remain incompletely understood. We investigated NK cell dysfunction following cryopreservation and evaluated DMSO-free cryoprotectant formulations as alternative strategies. DMSO-free formulations were optimized using a differential evolution algorithm. Cryopreserved NK-92 cells were assessed post-thaw for viability, recovery, proliferation, and cytotoxicity over 5 days. Raman cryomicroscopy and flow cytometry were used to characterize cytolytic granule localization and integrity during cooling and freezing. Pre-freezing chemical degranulation was performed to probe granule-mediated injury. Both DMSO-based and DMSO-free formulations had immediate post-thaw recovery and viability over 80% but had notable cell loss over 24 h. Although proliferation resumed by 48 h, expansion remained attenuated, and cytotoxic function was reduced by ≥ 20% and did not recover after 3–5 days. Imaging revealed temperature- and cryoprotective agent (CPA)-induced granule redistribution and destabilization. Pre-freeze degranulation improved short-term recovery and proliferation. These findings support cytolytic granule redistribution and destabilization as contributors to persistent NK cell dysfunction and may inform future cryopreservation strategies for NK cell therapies.

## Graphical abstract

**Figure:**
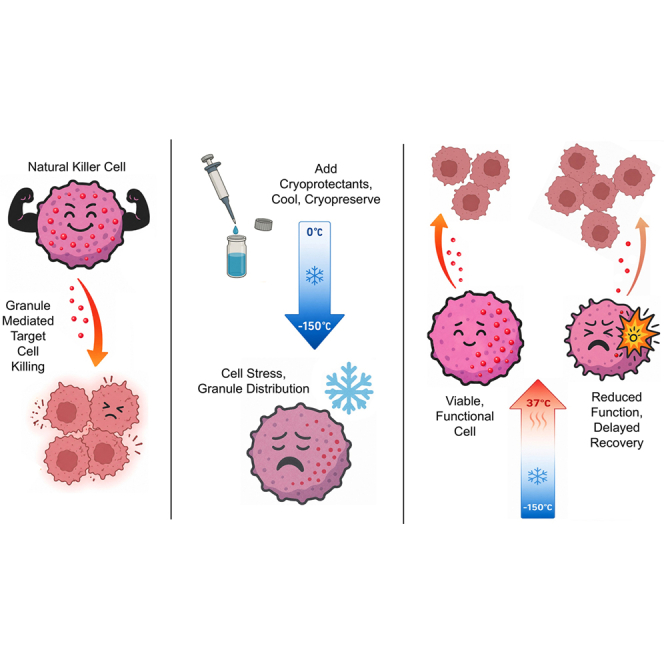

## Teaser

This study shows that natural killer cell cryopreservation can preserve their viability while impairing their longer-term cytotoxic function. The findings identify cytotoxic granule redistribution and destabilization within cells during cooling, cryoprotectant exposure, and freezing as contributors to post-thaw dysfunction, supporting preservation strategies that protect sustained target cell killing function.

## Introduction

Natural killer (NK) cells are powerful lymphocytes that have innate capabilities to target cancerous and infected cells while making up 5%–15% of the circulating lymphocytes.^1^^,^^2^ Unlike T and B cells, NK cells do not rearrange their receptor genes but rely on a variety of activating and inhibitory receptors (pattern recognition) to regulate cytotoxicity.^3^ NK cells recognize and eliminate virally infected or malignant cells without the need for prior sensitization or antigen presentation. Killing of target cells involves the formation of an immune synapse to eject cytolytic granules containing granzyme and perforin, while also producing key cytokines such as IFN-γ and TNF-α.^4^^,^^5^ Their broad tumor cell recognition ability, low risk of inducing graft-versus-host-disease (GVHD), and ability to be genetically modified make them a promising off-the-shelf candidate for immunotherapy.^6^

Cryopreservation is essential for transforming novel cell therapies into scalable, logistically feasible clinical products. It enables the storage of living cells over extended periods of time and transportation from the point of collection, manufacturing, storage, and eventual final location, referred to as the cold chain.^7^^,^^8^ Without cryopreservation, immunotherapies are restricted to patients close to both clinical and cell manufacturing sites, and infusions are scheduled in advance with little flexibility in timing. Cryopreservation would allow for the creation of large, stable cell banks to reduce manufacturing costs and transport off-the-shelf NK cell therapies to any hospital or clinic.^9^ Dry shippers enable shipping of cells at cryogenic temperatures, and liquid nitrogen dewars enable cryogenic storage, providing flexibility in treatment scheduling. Cold storage and cryopreservation are related preservation strategies but serve different roles in cell therapy manufacturing and delivery. Cold storage is generally used for short-term handling or transport, whereas cryopreservation at cryogenic temperatures enables long-term storage, wider distribution, and flexible clinical scheduling.

NK cells must remain viable and functional through sourcing, manufacturing, and infusion, making cryopreservation stability critical. NK cells, however, are particularly sensitive to cryopreservation, often showing reduced viability, impaired cytotoxicity, altered immunophenotypes, and poor *in vivo* persistence post-thaw compared with freshly expanded cells.^4^^,^^10^^,^^11^^,^^12^^,^^13^^,^^14^^,^^15^^,^^16^^,^^17^ 90% viability and recovery (defined as viable cell yield relative to pre-freeze) can be obtained immediately post-thaw, but viability and function degrade over time. This decline is especially pronounced within the first 24 h, and cell losses can be as high as 80%, affecting assays performed in this window.^12^^,^^15^^,^^18^ The extent of deterioration varies based on donor, cell density, cryomedium composition, freezing parameters, and prior expansion methods.^2^^,^^6^^,^^11^^,^^19^^,^^20^^,^^21^^,^^22^^,^^23^ Strategies such as resting cells in IL-2-supplemented media overnight have been reported to improve recovery and NK cell-induced cytotoxicity compared with cytokine-free media but lack the ability to fully restore *in vivo* function.^5^^,^^17^^,^^20^^,^^24^^,^^25^^,^^26^^,^^27^ Recent strategies of NK cell activation, immune cell engagers, and degranulation have shown promise in improving post-thaw performance.^1^^,^^15^^,^^28^^,^^29^ Still, current cryopreservation methods are limited in both effectiveness and scalability. Similar cryopreservation challenges have been observed in other innate immune cells, particularly granulocytes such as neutrophils, which are well recognized as difficult to cryopreserve with retention of post-thaw function despite preserved viability.^30^

Dimethyl sulfoxide (DMSO) has been the gold standard for biological material cryopreservation and is primarily used at a 5%–10% concentration range for mammalian cells.^31^ However, DMSO has well-known cytotoxic effects and clinical risks including allergic, gastrointestinal, neurological, and cardiac side effects.^32^^,^^33^^,^^34^^,^^35^ These adverse effects can limit the treatable patient population, especially limiting the treatment of glioblastoma, pediatric, and cardiac disease patients. Additionally, animal studies have demonstrated DMSO’s intrinsic neurotoxicity, with 2% DMSO intraventricular injection provoking hydrocephalus and astrocyte apoptosis^36^ and 1% DMSO intraventricular injection causing retinal ganglion cell death.^32^ DMSO-mediated cellular dysregulation, including epigenetic/mRNA changes at a 0.1% concentration, has also been reported, and it can affect cell function and stability over time, modify the cell cytoskeleton, downregulate the cell energy pathways, and reduce anti-apoptotic fitness.^37^^,^^38^^,^^39^^,^^40^ Despite its excellent cryoprotective properties, DMSO can lead to cell dysfunction and phenotypic changes, requiring DMSO to be added and removed quickly to avoid compounding damage to cells.^12^^,^^16^ These limitations have prompted efforts to reduce DMSO concentration or replace it altogether, though consistent and effective alternatives remain under development.

The development of an effective, non-toxic cryopreservation solution would be a major advancement in scaling out NK cell therapy, especially for sensitive treatment contexts such as glioblastoma, therapies requiring multiple infusions, or products requiring a large infusion volume. Although DMSO-containing cryopreservation methods remain clinically useful and can be feasible when products are washed prior to infusion or when infusion volumes are low, wash steps add processing complexity. They may also increase cell loss, cost, handling time, and reliance on clinical sites with appropriate cell-processing capabilities. Additionally, DMSO alternatives including sugars,^41^^,^^42^ polyols,^14^^,^^41^^,^^43^ amino acids,^38^^,^^44^ and a variety of polymers^14^^,^^45^^,^^46^^,^^47^^,^^48^ have been explored. Sugars demonstrate osmoprotective effects and stabilize plasma cell membranes.^49^^,^^50^ Sugar alcohols, glycerol in particular, displace intracellular water and stabilize protein structure by maintaining hydrogen bonding.^43^^,^^51^^,^^52^ Amino acids have demonstrated cryoprotection resulting in cell recoveries similar to DMSO and improved proliferation.^53^^,^^54^^,^^55^ Combinations of these components have also been optimized to cryopreserve multiple cell types such as T cells, mesenchymal stem cells (MSCs), induced pluripotent stem cells (iPSCs), and mixed cell populations. These components have been shown to form deep eutectic systems from high hydrogen bonding that reduce water activity, consequently reducing the amount of ice formation and stabilizing cells during cryopreservation.^53^^,^^56^^,^^57^^,^^58^

In this study, we optimized post-thaw recovery of NK cells preserved in osmolytes and evaluated post-thaw viability, proliferation, and NK cell-induced cytotoxicity over 5 days, using NK-92 cells as a model system to limit variation from donors with primary NK cells. Confocal Raman microscopy and flow cytometry were used to investigate activation and the osmotic and chilling damages as potential mechanisms of cell damage during freezing. NK cell recovery following activation-induced degranulation was compared to assess functional preservation across cryoprotectants.

## Results

### Optimization of the cryoprotective agent composition

**Figure 1:**
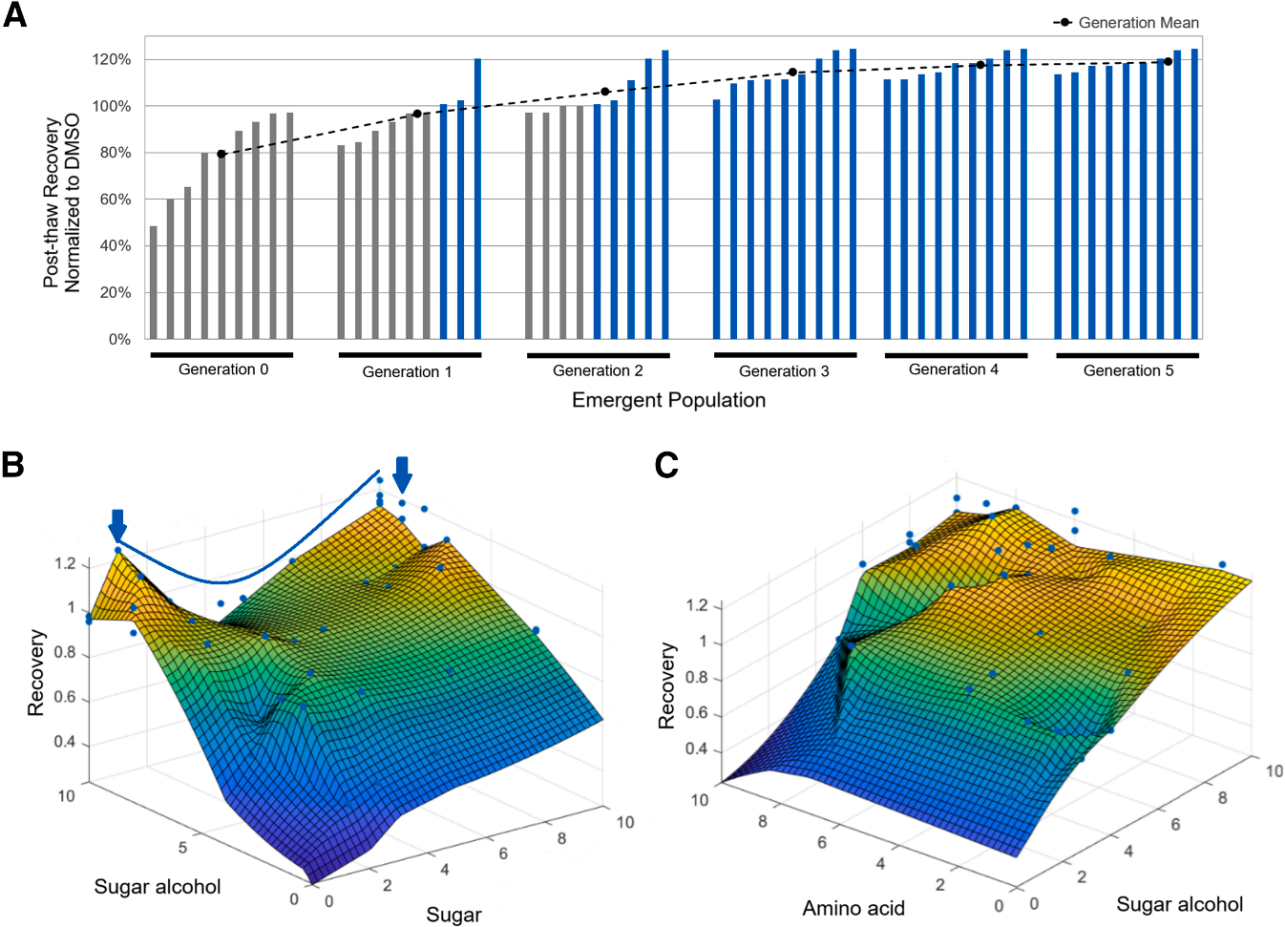
Differential evolution optimization of DMSO-free osmolyte cryopreservation solutions (A) Emergent population of algorithm-defined osmolyte compositions across five generations. Each column represents one candidate solution tested within a generation, and each solution contained different concentrations of sucrose, glycerol, and isoleucine. Nine formulations were tested per generation. NK-92 cells were cryopreserved in each formulation, thawed, and evaluated for post-thaw viable cell recovery normalized to the 10% DMSO control. Blue bars indicate formulations with recovery equivalent to or greater than the DMSO control, and the dashed line shows the generation mean. (B) Response surface showing post-thaw recovery as a function of sugar (sucrose) and sugar alcohol (glycerol) concentrations. Arrows point to the top-performing regions, and the curve was added to guide visualization. (C) Response surface showing post-thaw recovery as a function of amino acid (isoleucine) and sugar alcohol (glycerol) concentrations. *n* = 1 per formulation per generation.

The composition of a cryoprotective solution can play an important role in post-thaw cell survival. The first phase of this study used a differential evolution (DE) algorithm to optimize a three-component, DMSO-free osmolyte cryopreservation solution composed of sucrose, glycerol, and isoleucine. DE is an iterative optimization method in which candidate solutions are experimentally tested, and the resulting performance data are used to guide the next generation of candidate solutions. In this study, each candidate solution was defined by the concentrations of sucrose, glycerol, and isoleucine. A population size of nine was used, meaning that nine unique cryopreservation solutions were tested in each generation. Each column in Figure 1A represents one solution tested within a generation.

For each generation, NK-92 cells were cryopreserved in the nine algorithm-defined solutions, thawed, and evaluated for viable cell recovery. Recovery was normalized to the DMSO control and used as the optimization metric.^59^^,^^60^ The resulting recovery values were then provided back to the DE algorithm, which generated the next set of candidate solutions. Higher-performing solutions were retained in the emergent population, while new candidate formulations were generated to continue searching the composition space. This process was repeated until the algorithm converged, defined here as no further change in the emergent population between successive generations.

Three compositions with equivalent or better recovery than DMSO were found in the first generation shown in blue (Figure 1A). Across subsequent generations, the emergent population improved as the algorithm identified higher-performing regions of the composition space, including local and global maxima within the non-linear formulation landscape (Figures 1B and 1C). The algorithm converged in five generations. The best-performing solutions in the DE algorithm produced approximately 25% higher normalized recovery than the DMSO control. This phase was designed as an iterative optimization screen, rather than a statistical comparison of each individual formulation, which led to a sucrose:glycerol:isoleucine (SGI) solution for additional post-thaw characterization. High recovery was observed at high glycerol and both high and low levels of sucrose, while isoleucine concentration did not appear to affect recovery.

### Post-thaw characterization

**Figure 2:**
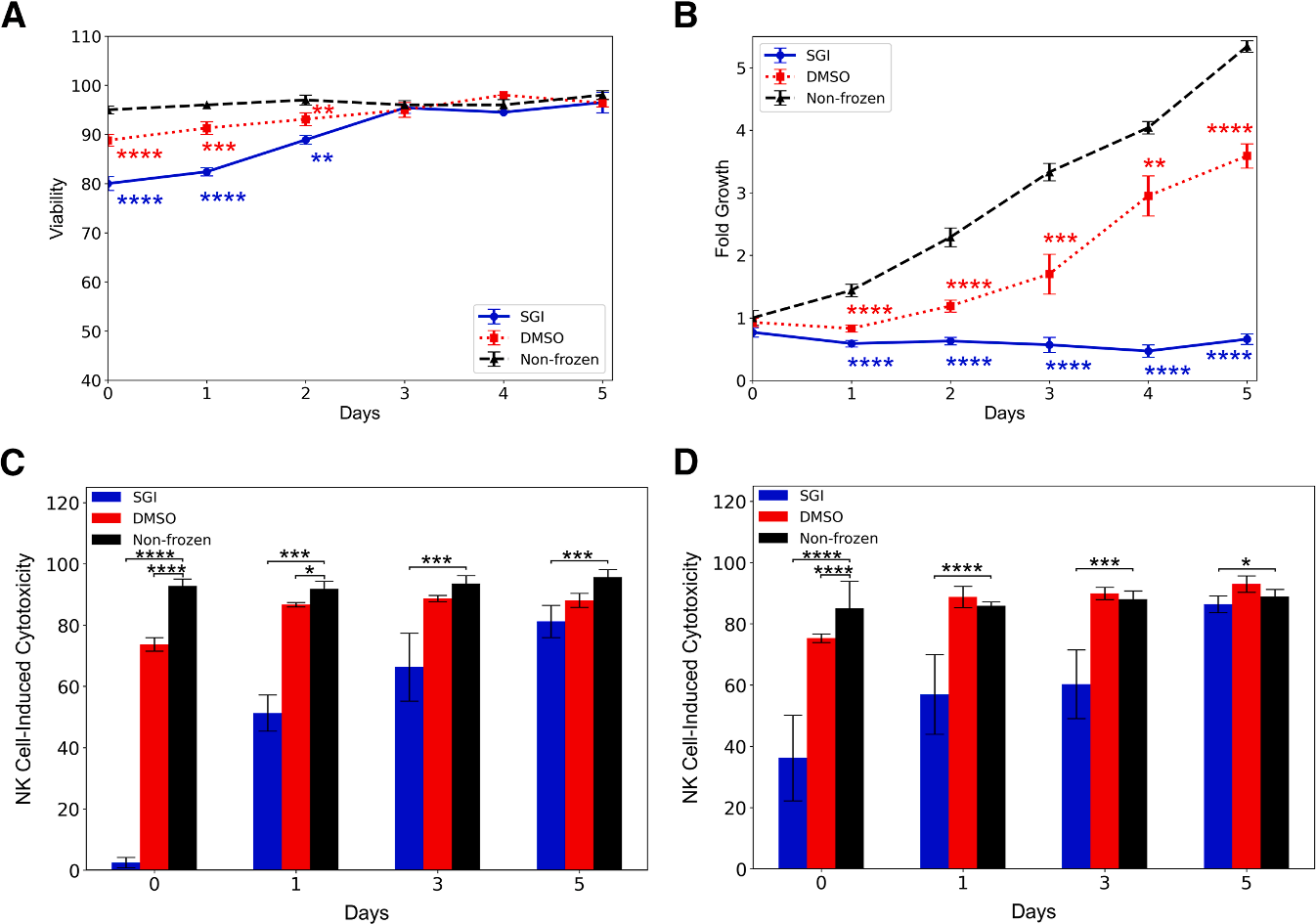
Cell persistence and cytotoxicity over 5 days post-thaw Viability (A) and recovery (B) of thawed NK cells frozen in the top DE algorithm solution SGI or DMSO were measured over 5 days post-thaw. Recovery was calculated as viable cell number normalized to the number of viable cells prior to freezing. (C and D) NK cell-induced cytotoxicity at E:T ratios of 4:1 (C) and 2:1 (D) was determined by harvesting the cells at days 0, 1, 3, and 5 after thawing and co-culturing with K562-GFP target cells for 24 h. Cytotoxicity was calculated from the change in target cell number relative to target-only controls. Pairwise statistical comparisons were performed between DMSO and SGI at each time point, using two-tailed Student’s *t* tests. Error bars indicate standard deviation; *n* = 4. Significance is indicated as ∗*p* < 0.05, ∗∗*p* < 0.01, ∗∗∗*p* < 0.005, and ∗∗∗∗*p* < 0.001.

Cell viability, recovery, proliferation, and cytotoxicity were evaluated over 5 days post-thaw (Figure 2) for the top-performing multi-component osmolyte solution and a DMSO-containing control. Immediately post-thaw, both SGI- and DMSO-preserved NK-92 cells maintained high viability (Figure 2A). However, post-thaw recovery and persistence differed over time (Figure 2B). SGI-preserved cells showed strong immediate recovery but reduced expansion over the 5-day recovery period compared with DMSO-preserved cells. This demonstrates that improved immediate post-thaw recovery did not necessarily translate into improved longer-term proliferation or persistence.

NK cell-induced cytotoxicity was evaluated by culturing thawed cells and harvesting NK-92 cells at days 0, 1, 3, and 5 for co-culture with K562 green fluorescent protein (GFP) target cells at effector-to-target cell (E:T) ratios of 4:1 and 2:1 (Figures 2C and 2D). Cells frozen in both solutions had significantly reduced cytotoxicity compared with non-frozen NK-92 cell controls. Although cytotoxicity partially recovered during post-thaw culture, SGI-preserved cells showed reduced functional recovery compared with DMSO-preserved cells, particularly at earlier time points and at the 4:1 E:T ratio. These results indicate that SGI improved immediate recovery in the optimization screen but did not fully preserve post-thaw proliferation or cytotoxic function. The largest increases in cytotoxicity were observed at day 1 after recovery in culture and between days 3 and 5 with a medium change performed after day 3. NK cells cryopreserved in DMSO did not recover function at the 4:1 E:T ratio until 3 days post-thaw and had a slower proliferation rate than non-frozen cells. This demonstrates that it takes 5 days for the killing action of the cells in osmolyte-free solutions to recover. The next phase of the paper describes using studies to understand the influence of the cryopreservation steps on the cytolytic granules of the NK cells.

### NK cell granule activation

**Figure 3:**
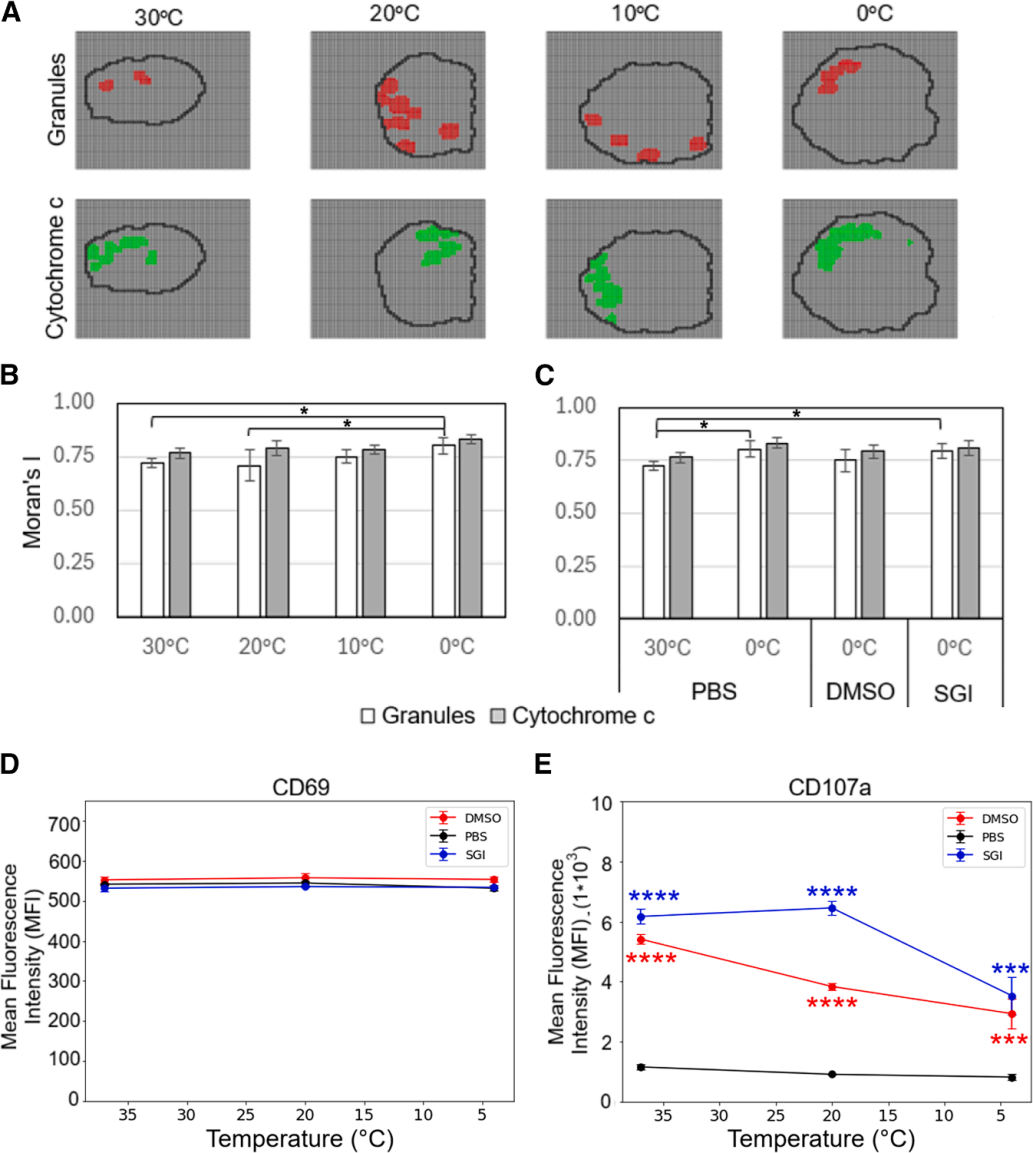
Temperature- and CPA-associated changes in NK cell granule localization (A) Representative Raman false-color images of NK cells chilled in PBS, highlighting solution (gray, 3,125 cm^−1^), cell boundary (black, 1,660 cm^−1^), carotenoid/granule (red, 1,525 cm^−1^), and cytochrome *c*/mitochondria (green, 1,127 cm^−1^). (B and C) Moran’s I quantification of granule and mitochondria spatial localization or clustering for chilled NK cells in PBS (B) and CPA solutions (C); higher positive values indicate greater spatial localization or clustering of signal. Granules had significantly (*p* < 0.05) higher Moran’s I after chilling from 30°C to 10°C and 0°C, indicating greater localization, and Moran’s I was significantly (*p* < 0.05) higher for chilled cells in SGI. (D and E) Mean fluorescence intensities (MFIs) of CD69 (D) and CD107a (E) of NK cells incubated in DMSO, PBS, or SGI at temperatures from 37°C to 4°C. Error bars indicate standard deviation; *n* = 10 (for Raman imaging) and 3 (for flow cytometry). Significance is indicated as ∗*p* < 0.05, ∗∗∗*p* < 0.005, and ∗∗∗∗*p* < 0.001.

Previous studies have demonstrated that post-thaw function and persistence of NK cells are poor and that the granules play a role in freezing damage and post-thaw cell losses.^1^^,^^4^^,^^15^ The next phase of the investigation involved using low-temperature Raman spectroscopy to interrogate the cells at low temperatures. The cells were resuspended in phosphate buffered saline (PBS), pipetted onto a Peltier stage, cooled to target temperatures of 30°C, 20°C, 10°C, and 0°C (Figure 3A), and held for 15 min. The granules were imaged (Figure 3) in the cell based on the Raman signature for carotenoids (1,525 cm^−1^), which is associated with granules.^61^^,^^62^ As temperature decreased below 30°C, the distribution of granules changed, and they appeared to migrate toward the periphery of the cell and closer to the plasma membrane.

The change in spatial distribution was quantified using Moran’s I, a measure of spatial autocorrelation. Moran’s I ranges from −1 to +1, where −1 indicates perfect dispersion of signal, 0 indicates a random distribution, and +1 indicates strong spatial localization or autocorrelation. Because cytolytic granules are discrete organelles, granule-associated Raman signal was expected to fall within the positive range. Therefore, an increase in Moran’s I for granules would indicate that the granule-associated signal became more spatially localized or clustered within the cell.

As NK cells were cooled from 30°C to 0°C, the Moran’s I for the granules increased from 0.72 to 0.80 (*p* < 0.05, Figure 3B). Although this numerical change appears modest, Moran’s I is constrained between −1 and +1, and the baseline signal is already positive because granules are localized organelles. Thus, further increases indicate a measurable shift toward greater granule localization or clustering during cooling.

As most cryopreservation protocols involve both cooling and the introduction of a cryoprotective agent (CPA), the cells were also imaged after introducing CPA solutions and chilling to 0°C (Figure 3C). NK cells exposed to DMSO and chilled to 0°C did not have a significantly different Moran’s I (0.75) from that of cells at 30°C (0.72). In contrast, the cells exposed to the osmolyte solutions (SGI) and cooled to 0°C had a significantly higher granule Moran’s I (0.80), suggesting greater granule localization or clustering during combined CPA exposure and chilling.

The mitochondria have been identified as a potential site of damage during freezing for other immune cells.^63^ A similar analysis was performed to quantify the distribution of cytochrome *c* (Table 2, 1,127 cm^−1^), using the same Raman images analyzed for distribution of the granules. The distribution of cytochrome *c* is a cell viability metric where leakage or loss of cytochrome *c* indicates a dead or damaged cell.^63^ A change in the cytochrome *c* distribution may also indicate an activation of the NK cell or a change in cell health. Under exposure to both reduced temperatures and CPAs, the distribution of cytochrome *c* remained unchanged.

### Expression of cell surface markers

The low-temperature Raman images suggest that the cells may be activated by low temperatures and exposure to CPAs. To characterize the extent of that activation, NK cells were subjected to different temperatures for 1 h and stained for CD69, CD107a, NKG2D, and CD56 to determine whether cell surface expression is altered as well. There was no change in CD69 mean fluorescence intensity (MFI) with temperature (Figure 3D), and expression was less than 12%. There was also no change in the MFI of CD69 with exposure to PBS, DMSO (CryoStor CS10), or SGI. The MFI for CD107a (Figure 3E) was unchanged under exposure to PBS across temperatures but was significantly higher under exposure to DMSO and osmolytes at 37°C and 20°C. Expression of CD107a was approximately 10% for PBS and as high as 82% and 78% for SGI and DMSO, respectively. However, CD107a MFI was 3.5 × 10^4^ for the highest phorbol-12-myristate-13-acetate (PMA) and ionomycin concentrations in the 24-h dosing study, described in the following section, whereas the highest MFI was 6.3 × 10^3^ for SGI, a 5.4-fold difference. The MFIs of CD56 and NKG2D were also measured but did not significantly change with temperature or exposure to CPA solution over the durations tested.

### Pre-freeze degranulation

**Figure 4:**
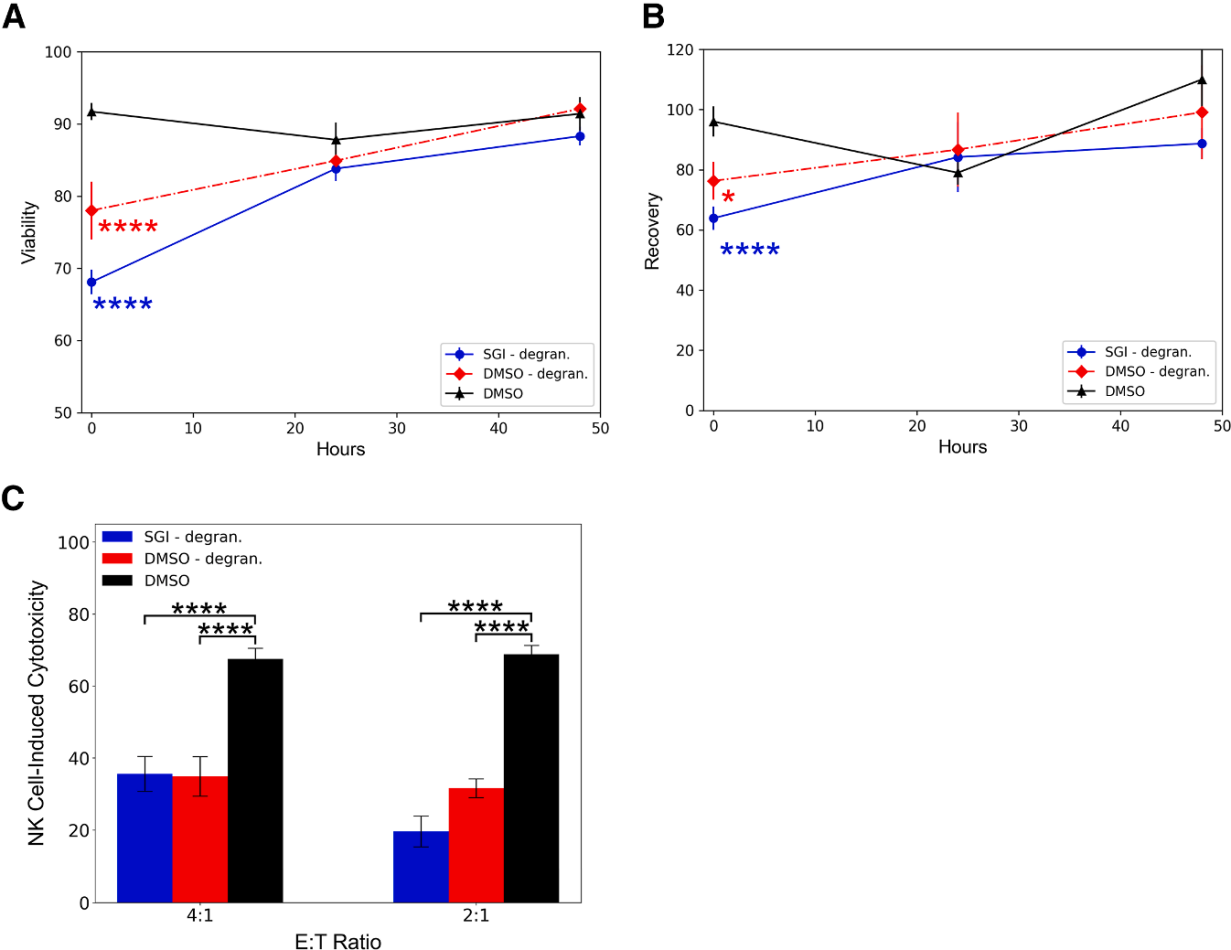
Degranulated NK cell viability, recovery, and cytotoxicity post-thaw Viability (A) and recovery (B) of degranulated, frozen, and thawed NK cells were measured over 48 h. (C) NK cell-induced cytotoxicity of degranulated, frozen, and thawed NK cells was determined after 24 h in co-culture. Immediate post-thaw viability was significantly (*p* < 0.0001) lower for degranulated cells, and post-thaw recovery was significantly lower for degranulated cells in DMSO (*p* < 0.05) and SGI (*p* < 0.0001). Post-thaw cytotoxicity was significantly (*p* < 0.0001) reduced for degranulated NK cells compared with untreated NK cells frozen in DMSO. Error bars indicate standard deviation; *n* = 4. Significance is indicated as ∗*p* < 0.05, ∗∗∗*p* < 0.005, and ∗∗∗∗*p* < 0.001.

A recent study demonstrated that degranulation of the cells prior to freezing improved NK cell post-thaw recovery and persistence.^1^ To see if a similar outcome was observed using the solutions and freezing conditions for this study, NK cells were cultured for 24 h with PMA and ionomycin in order to induce degranulation and then assessed for viability and yield. Percent degranulation was measured by CD107a expression, using flow cytometry, compared with that of non-treated cells. Exposure to PMA and ionomycin had a negative effect on the cells, with viability significantly decreasing for all doses tested and ranging from 73% to 87% compared with 95% for a non-treated control. Cell yield from the 24-h degranulation treatment ranged from 83% to 100%, while the non-treated cells yielded 168%, indicating a significant reduction in proliferation. A series of experiments were performed using 5 ng/mL PMA and 50 nM ionomycin to degranulate NK cells for 24 h before freezing in multi-component osmolyte solutions (Figure 4). This condition was chosen based on obtaining high degranulation (88%) and high yield (87%), with viability remaining above 80% after treatment. The degranulated cells were frozen and thawed; post-thaw viabilities and recoveries ranged from 63% to 78% (68% excluding degranulated CS10) and 67% to 87% (or 77% not including degranulated CS10), respectively. This post-thaw recovery also includes the 13% cell loss associated with degranulation.

Thawed cells were plated for a cytotoxicity assay, and remaining cells were cultured for 48 h. For all solutions, degranulated cells proliferated in the first 24 h in the post-thaw culture, gaining 14% for CS10 and up to 38% for osmolyte solutions, with non-degranulated NK cells frozen in DMSO proliferating over 48-h reflecting a recovery over 100% compared with the number of viable cells pre-freezing. This contrasts with non-degranulated cells frozen in CS10, which exhibited a 17% cell loss primarily due to apoptosis. Viability increased significantly after 24 and 48 h in culture for degranulated cells, which had equivalent viability to the non-degranulated cells frozen in DMSO. Cytotoxicity over the first 24 h post-thaw was negatively impacted where degranulated NK cells had significantly reduced cytotoxicity compared with non-degranulated NK cells frozen in DMSO.

### CPA penetration into granules

**Figure 5:**
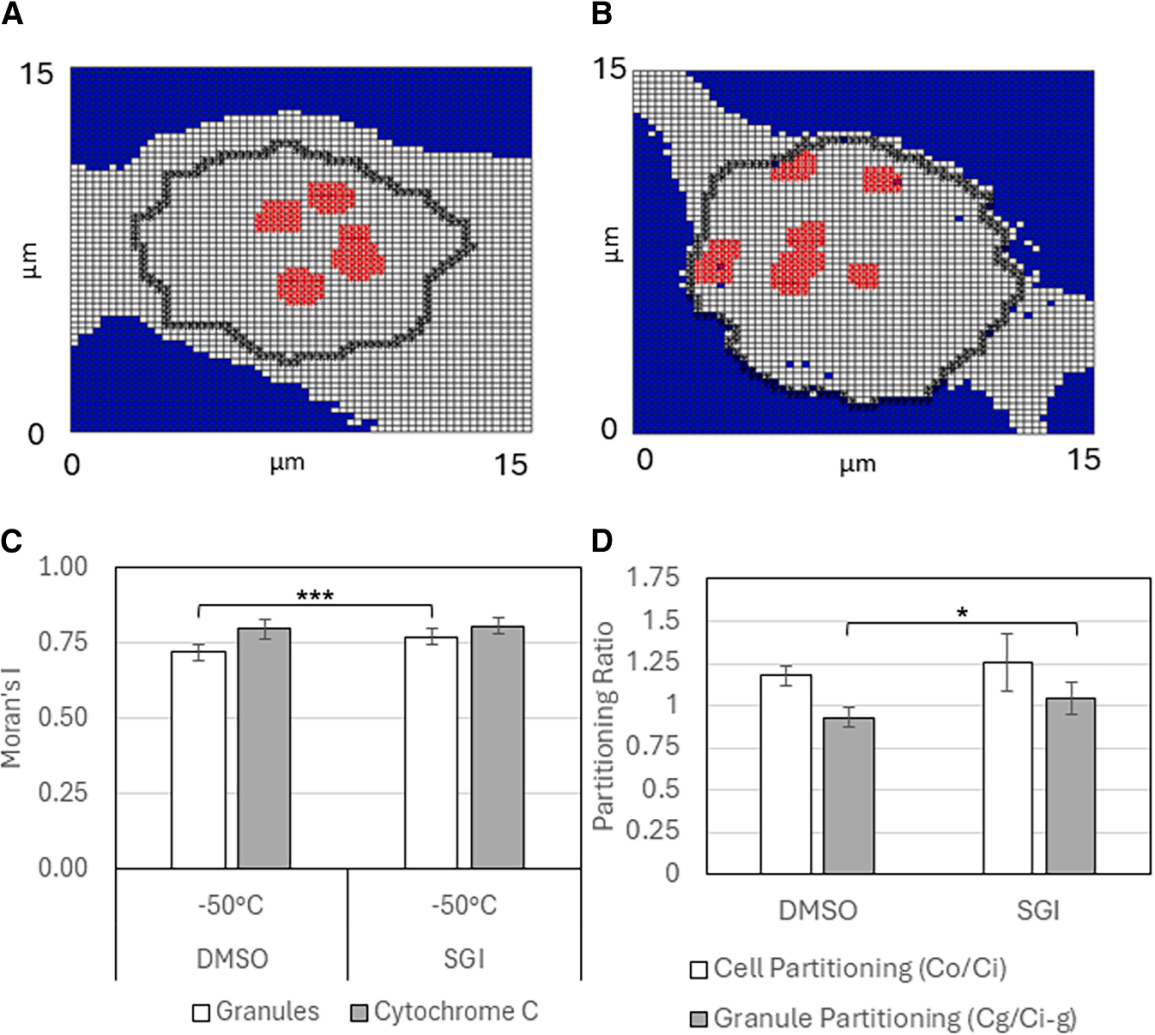
Raman confocal microscopy of frozen NK cells Representative Raman false color images of single NK cells frozen in osmolytes (A, SGI) and DMSO (B) overlaid with ice (blue), cell boundary (black), non-ice (gray), and granules (green, 1,525 cm^−1^). (C) Spatial localization of granules and cytochrome *c* in frozen NK cells was quantified with Moran’s I. Granule Moran’s I was significantly (*p* < 0.001) higher for cells frozen in SGI. (D) Analysis of cryoprotectant partitioning into the cell (C_o_/C_i_) and intracellular cryoprotectant partitioning into granules (C_g_/C_i-g_). The CPA partitioning ratio from the intracellular space into granules was significantly higher (*p* < 0.05) for cells frozen in SGI than for those in DMSO. Error bars indicate standard deviation; *n* ≥ 7. Significance is indicated as ∗*p* < 0.05 and ∗∗∗*p* < 0.005.

The studies described above demonstrated changes in the distribution of granules when cooled down or exposed to CPAs. The freezing process may also influence granule distribution within cells. Similar Raman imaging methods were used to analyze granule distribution by freezing NK cells to −50°C in SGI or DMSO (Figures 5A and 5B). The spatial localization of granules and cytochrome *c* (Table 2, 1,127 cm^−1^) was evaluated with Moran’s I (Figure 5C). Granules in the NK cells frozen with osmolytes had a significantly higher Moran’s I than the NK cells frozen with DMSO, which indicated greater granule localization or clustering in the osmolyte solutions. In addition, the integrity of mitochondria was determined using cytochrome *c* signal. No difference in cytochrome *c* for frozen cells was observed compared with cells at room temperature. Moran’s I was not significantly different for cells frozen in DMSO or osmolytes (Figure 5C), suggesting that the freezing conditions tested did not produce detectable cytochrome *c* redistribution.

CPA solutions are hypertonic and when they are introduced, there can be significant changes in the volume resulting from an efflux of water followed by an influx of CPA.^64^ In addition, the distribution of CPA in cells is important and may influence the stability of subcellular structures such as granules. Low-temperature Raman spectroscopy can be used to quantify the distribution of CPA inside cells and characterize the partitioning of CPA. A metric for quantifying the distribution CPA is the partitioning ratio, which is the ratio of average non-ice cryoprotectant intensity outside the cell to the average cryoprotectant intensity inside the cell and is represented as follows:

(Equation 1) P=CoCi

where C_o_ is the average intensity of cryoprotectant outside the cell in the non-ice region, and C_i_ is the average intensity of the cryoprotectant inside of the cell. The partitioning ratio for NK cells frozen in DMSO was 1.18, and the partitioning ratio for NK cells frozen using osmolytes was 1.26 (Figure 5D).

The penetration of CPAs into the granules is also important. Poor or incomplete penetration of CPA into granules may be one potential mechanism by which NK cells are damaged during freezing. Raman spectra from pixels fully within a granule were compared with those from other intracellular pixels, and a partitioning ratio for the granule was calculated based on the following equation:

(Equation 2) Pg=CgCc

where C_g_ is the average intensity of cryoprotectants inside the granule, and C_c_ is the average intensity of cryoprotectants inside the cell not including the granules. Spectra obtained demonstrated that DMSO and glycerol were present in NK granules at −50°C, with granule partitioning ratios being 0.93 for NK cells frozen with DMSO and 1.04 for NK cells frozen with osmolytes. These results demonstrate that CPAs were present in granules at levels comparable to the surrounding cytosol and did not affect the distribution of mitochondria. Together with Figure 3, these data suggest that the reduced function observed after cryopreservation with osmolytes is unlikely to be explained solely by the exclusion of CPA from granules. Instead, altered granule localization during chilling and freezing may be a contributing mechanism.

### Sugar alcohol osmotic stress

**Figure 6:**
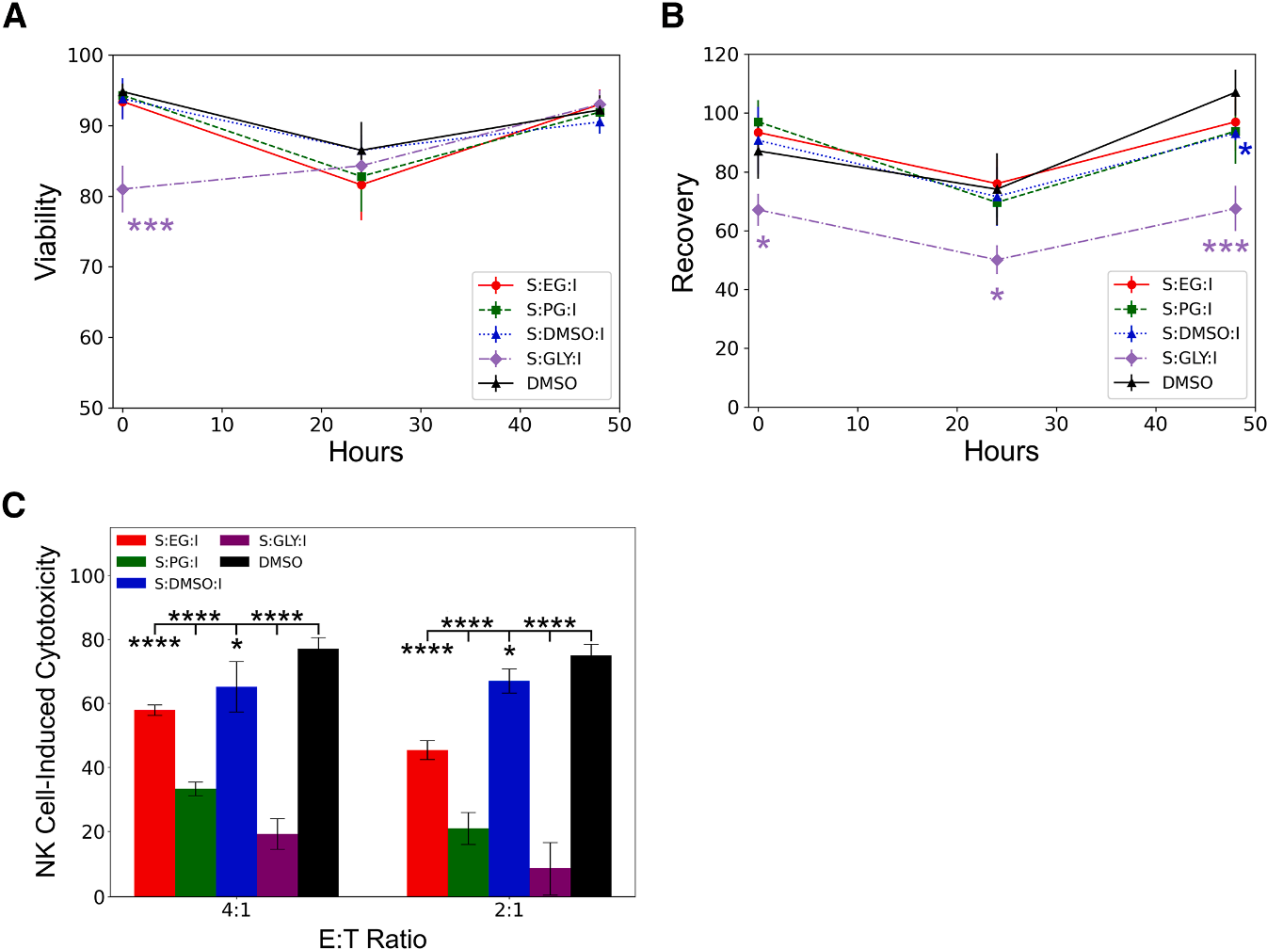
Influence of sugar alcohol molecular weight on post-thaw performance Glycerol was swapped for other sugar alcohols, and viability (A) and recovery (B) of thawed NK cells were measured over 48 h. (C) Thawed NK cell-induced cytotoxicity was determined after 24 h in co-culture. Immediate post-thaw viability and recovery were significantly (*p* < 0.0001) lower for S:GLY:I. Post-thaw cytotoxicity was significantly (*p* < 0.0001) lower for all solutions than for DMSO except S:DMSO:I. EG, ethylene glycol; PG, propylene glycol; DMSO, dimethyl sulfoxide; GLY, glycerol; S, sucrose; I, isoleucine. Error bars indicate standard deviation; *n* = 3–4. Significance is indicated as ∗*p* < 0.05, ∗∗∗*p* < 0.005, and ∗∗∗∗*p* < 0.001.

Previous studies performed in our lab suggest that NK cells are osmotically sensitive.^10^ Glycerol is a high-molecular-weight sugar alcohol that enters the cell through passive diffusion through the cell membrane. Lower-molecular-weight CPAs tend to diffuse more readily through the cell membrane, resulting in lower osmotic stresses to the cell. To test the influence of osmotic stress on cell function, glycerol was swapped with another sugar alcohol (ethylene glycol, propylene glycol, and DMSO) at an equivalent millimolar concentration and following the same methods described. The top differential evolution osmolyte solution was taken as a reference, and glycerol was swapped for ethylene glycol (62 g/mol), propylene glycol (76 g/mol), or DMSO (78 g/mol). Post-thaw viability and recovery were evaluated over 2 days post-thaw (Figures 6A and 6B), and cytotoxicity was evaluated immediately post-thaw (Figure 6C). Post-thaw viability and recovery were indistinguishable between the different solutions tested (DMSO and osmolyte solution with different sugar alcohol components when substituting equivalent millimolar concentrations of sugar alcohols after thawing), except for glycerol (92 g/mol), which had a significant drop and remained low through 48 h. NK cell-induced cytotoxicity post-thaw trended lower with greater sugar alcohol molecular weight. The exception was using DMSO (78 g/mol) in the cryopreservation solution that performed significantly better than propylene glycol.

## Discussion

Despite using only three components and a single outcome measure, the DE algorithm revealed a complex response topology, with top-performing formulations observed at both low and high sucrose concentrations. This complex topology of the CPA composition with global and local maximums has been observed with multiple cell types, adherents, and suspensions. Non-linear post-thaw recovery with composition has been observed in Jurkat T cells, mesenchymal stromal cells, iPSCs, and cardiomyocytes.^44^^,^^59^^,^^65^ Studies with other approaches and components have also shown complex interactions with and without DMSO.^42^^,^^66^^,^^67^^,^^68^^,^^69^ This further emphasizes that higher concentrations of CPAs do not directly correlate with the higher post-thaw performance.

Although immediate post-thaw viability is commonly used to assess cryopreservation success, the cells must also function and persist after thawing. The difference between immediate recovery and longer-term functional performance highlights an important limitation of using post-thaw viability or recovery as the sole measure of cryopreservation success. This phase of the investigation looked at the rescue of function and proliferation over multiple days beyond immediately post-thaw or just after an overnight rest. SGI-preserved NK-92 cells showed strong immediate recovery, but this did not translate into equivalent proliferation or cytotoxic function compared with DMSO-preserved cells.

This type of disconnect between apparent cell survival and functional recovery has been reported for other cryopreserved cell types.^70^ For example, Page noted that colony-forming unit was a strong predictor for CD34^+^ cell engraftment, and Lysak noted cases of engraftment failure despite high post-thaw viable cell counts.^71^^,^^72^ Similar findings have been observed in hepatocyte monolayers where Tomás and Stéphenne found reduced metabolic activity and cell plating attachment despite cells frozen with acceptable viability.^73^

For NK cells, decreased cytotoxicity after thawing was observed previously by Mark and colleagues.^4^ Granule-associated injury may be one contributor to this functional impairment as damaged granules could release cytolytic contents inside cells causing cell stress, apoptosis, or loss of function. Berjis et al. recently showed that partial degranulation and upregulation of anti-apoptotic pathways can improve post-thaw NK cell outcomes, supporting the idea that granule-associated injury and delayed cell death pathways are important features of NK cell cryoinjury.^1^

In this work, SGI-cryopreserved cells persisted after thawing but exhibited impaired proliferation and cytotoxicity, suggesting that the formulation preserved short-term recovery more effectively than longer-term effector function. These studies demonstrated that a freezing protocol with immediate post-thaw recovery and viability could be developed, and although those cells persisted, killing function was impaired. The functional impairment may be due to a variety of mechanisms including transcriptional changes, granule redistribution, cytoskeletal disruption, osmotic stress, delayed apoptosis, metabolic changes, or incomplete restoration of cytotoxic machinery after thawing. Exposure to a CPA or supplement can negatively impact cell function, even with high post-thaw recovery after thawing.^10^ Delayed-onset apoptosis can result in a loss of viable cells, especially in the first 24 h after thawing, and temporarily slows proliferation. Studies have demonstrated that the addition of IL-2 post-thaw can improve killing function of the cells, but additional work is needed to determine which strategies best preserve both recovery and sustained function.^1^^,^^20^^,^^26^^,^^74^^,^^75^ The improved immediate recovery but reduced proliferation and cytotoxicity observed with SGI suggests that the formulation preserved short-term viable cell yield more effectively than longer-term effector function. This trade-off may reflect delayed apoptosis, osmotic stress during CPA exposure, altered granule trafficking or degranulation pathways, metabolic stress, mitochondrial dysfunction, or incomplete recovery of cytotoxic machinery after thawing. Future studies using transcriptomic, proteomic, metabolic, and cytoskeletal analysis could help distinguish where the damage has the longest-lasting impact.

The results of this investigation support the premise that granules play an important role in the post-thaw persistence and function of NK cells.^1^^,^^4^ The change in the distribution of granules observed using low-temperature Raman spectroscopy is consistent with granule convergence toward the microtubule-organizing center (MTOC).^76^ This process may indicate NK cell activation or early degranulation, suggesting that sub-physiological temperatures or cryoprotectant exposure could inadvertently trigger activation. Importantly, these changes occurred during cooling and CPA exposure without target-cell stimulation. This suggests that cryopreservation-relevant stress may induce activation-like granule redistribution, although additional mechanistic studies are needed to determine whether this redistribution directly causes post-thaw functional impairment.

The Moran’s I results provide a quantitative way to interpret changes in organelle-associated Raman signal during cooling, CPA exposure, and freezing. Moran’s I changes within an already positive range can appear numerically modest but still indicate meaningful changes in spatial organization. In this study, increased granule Moran’s I during cooling, SGI exposure, and freezing indicates greater granule localization or clustering during cryopreservation-relevant stress.

Prior Raman-based studies using Moran’s I to evaluate cytochrome *c* localization during intracellular ice formation provide a useful comparison.^63^ In viable cells, cytochrome *c* remains localized to mitochondria and produces higher Moran’s I values, whereas intracellular ice formation and mitochondrial damage reduce cytochrome *c* localization and shift Moran’s I toward lower values. By analogy, if cytolytic granule membranes were broadly disrupted and granule contents became diffuse, granule-associated Moran’s I would be expected to decrease toward a more random distribution. Instead, the higher Moran’s I suggests that cooling in SGI exposure promoted granule localization or clustering rather than loss of granule-associated signal.

The biological importance of this change is that cytolytic granules are not passive structures; they are central to NK cell target killing and normally undergo regulated trafficking during immune synapse formation and degranulation. Therefore, redistribution or clustering of granules during cooling, CPA exposure, or freezing may indicate unintended activation-like behavior or altered granule trafficking during the cryopreservation process. The lack of corresponding cytochrome *c* redistribution suggests that these conditions did not produce broad mitochondrial disruption detectable by Raman microscopy but instead produced a more granule-associated spatial response. Together, these findings support a model in which cryopreservation-relevant stress alters cytolytic granule organization, which may contribute to reduced post-thaw NK cell function.

For cells chilled to 0°C, there was no difference in granule distribution for NK cells in PBS or DMSO, but there was a significant increase in Moran’s I for NK cells held in osmolyte solution at 0°C. This indicates that NK cells show greater signs of activation at lower temperatures when held in osmolytes compared with those when held in DMSO, which may be a contributing factor for why post-thaw cytotoxicity is lower for cells frozen in osmolytes.

Chilling injury has been reported in other cell types including platelets, neutrophils, sperm, and oocytes but has been less studied in NK cells.^77^^,^^78^^,^^79^ Earlier studies reported that short-term (≤24 h) storage at 4°C did not impair NK cell cytotoxicity, but significant functional loss occurred after cryopreservation, possibly due to granule damage rather than chilling injury.^78^^,^^80^ In addition, NK cell stress can induce ADAM17-mediated receptor shedding, leading to cleavage of CD16a, TNF receptors, and adhesion molecules amplifying activation signals and potentially confounding post-thaw assays.^81^

Cytochrome *c* is a mitochondrial stress and apoptosis marker and could reflect intracellular content redistribution or damage. For example, cytochrome *c* release was detected after DMSO exposure to mesenchymal stromal cells and sensory neuronal cells.^53^^,^^82^ However, no change in cytochrome *c* was detected for NK cells chilled to 0°C in PBS, DMSO, or osmolytes. This suggests that neither the mitochondria were damaged nor did the NK cells become apoptotic from cooling, but granules were mobilized. Mitochondrial health can impact NK cell immune function, and NK cell activation can alter glycolysis and mitochondrial respiration.^83^ There is limited work on the direct impact of cryopreservation on mitochondrial potential or reactive oxygen species (ROS) for NK cells, though work by Zhang et al. demonstrated increased mitochondrial ROS in CD4 T cells after cryopreservation.^84^

### Expression of cell surface markers

CD107a expression was higher for NK cells in CPAs and at higher temperature but not for the cells in PBS. The shrinkage of cells from being in a hypertonic solution could squeeze and fuse granules with the cell lipid bilayer. The glycerol in SGI is slower to penetrate cells than DMSO due to its lower molecular weight. This results in a greater volume change as water rushes out of the cell, but glycerol is slower to enter. At 4°C, the mechanisms involved in granule trafficking may be slowed enough that fusion did not occur within 1 h.

CD107a expression generally increases after cryopreservation as well, likely due to the freezing process itself, rather than target cell exposure, with CPA exposure also contributing to this effect.^85^ While some studies have observed elevated CD107a expression after co-culture with K562 cells post-thaw,^85^ others have reported no significant change when using different CPAs such as DMSO, glycerol, or ethylene glycol, whether co-cultured with target cells or stimulated with cytokines.^12^^,^^14^^,^^15^^,^^86^ Notably, one study required baseline adjustment due to non-specific degranulation from the freeze-thaw process, despite no change observed with target exposure.^12^ CD69 expression appears more variable post-thaw and protocol dependent.^6^ Some studies have reported a decline compared with fresh cells, though this was reversible after a 16-h rest, while others have observed upregulation during the post-thaw recovery phase, correlating with an increased antibody-dependent cellular cytotoxicity ADCC activity.^15^^,^^87^

### Pre-freeze degranulation

A significant challenge in NK cell cryopreservation is intracellular damage caused by the leakage of granzyme B (GZMB) protein from cytolytic granules.^1^ When granules are disrupted during freezing and thawing, released GZMB induces apoptosis, substantially reducing NK cell viability and function post-thaw. One proposed approach to address this problem is the removal of these cytolytic payloads prior to cryopreservation by preemptive degranulation, aiming to prevent or minimize this damage mechanism altogether. We tested a chemical-based approach to degranulate NK cells by using a combination of PMA and ionomycin. This stimulation bypasses cell-surface receptor interactions, activating intracellular signaling pathways pharmacologically. Cryopreserved, PMA/ionomycin-treated NK cells proliferated in the first 24 h post-thaw, unlike non-degranulated cells, but this treatment did not markedly enhance NK cell-induced cytotoxicity. Low cytotoxicity immediately post-thaw is expected as the cells need to regenerate granules, and little to no proliferation in the first 24 h is likely to occur from the degranulation step itself.

Our findings align with those of Berjis et al. who reported that partial degranulation using a cytokine cocktail (IL-18 and IL-15) provided better post-thaw outcomes than pharmacological stimulation with PMA/ionomycin.^1^ The authors postulated that the primary benefit arose from anti-apoptotic effects, notably through the upregulation of BCL2L1 expression at both RNA and protein levels, effects further enhanced by IL-15 co-treatment. These results underscore that degranulation alone may not sufficiently protect NK cells. Other groups have shown higher CD107a expression, viability, proliferation, and activating receptor expression with stimulation to cytokines.^88^^,^^89^^,^^90^ Cytokine-based priming with IL-15 and IL-18 may provide a more physiologic approach than PMA/ionomycin stimulation by coupling partial degranulation with survival, metabolic, and anti-apoptotic signaling. Future studies should determine whether IL-15/IL-18 priming alters granule localization during chilling or CPA exposure and whether allowing a post-thaw recovery period improves cytotoxic function after pre-freeze degranulation.

Supporting this concept, Oyer et al. demonstrated the importance of pre-cryopreservation priming strategies that engage protective cellular mechanisms.^15^ Oyer’s use of membrane-bound IL-21 (mbIL21)-activated NK cells eliminated the typical dependence on post-thaw IL-2 supplementation, suggesting beneficial alterations to cellular metabolism or anti-apoptotic pathways. Oyer also observed transient reductions in activation markers such as CD69, GZMB, and perforin immediately post-thaw, which recovered after 16 h of rest, correlating with restored cytotoxic function. These observations indicate that pre-freeze priming can mitigate immediate post-thaw damage, although the underlying mechanism may involve more than granule removal.

Osmotic stress has long known to affect cell viability from rapid water intake or removal, activating various pathways in cell stress response, proliferation, and apoptosis.^91^^,^^92^^,^^93^ The 60-min CPA exposure used in this study was intended to allow equilibration prior to freezing. However, CPA exposure itself may contribute to cellular stress independent of freezing. Separate hold-time studies suggested that this exposure duration was not sufficient to drive any pre-freeze viability loss in NK-92 cells (unpublished data). For NK cells frozen to −50°C in osmolytes and DMSO, there was a significant increase in granule Moran’s I for cells frozen in osmolytes relative to freezing in DMSO. However, these values were not significantly different from Moran’s I values for NK cells held at 30°C in PBS. Cytochrome *c* distribution was also unchanged, indicating that membrane integrity is not compromised during the cryopreservation process.

Previous studies have shown that the partitioning ratios closer to 1 result in higher recoveries to multiple cell types.^10^^,^^65^^,^^94^ The partitioning ratio of NK cells frozen in DMSO were closer to 1 compared with the partitioning ratio of NK cells frozen in osmolytes, but the difference was not statistically significant. The partitioning of CPA inside the cells is like that of the concentration of CPAs inside the granules for both osmolytes and DMSO cryoprotectants for NK cells, suggesting that it is the activation of granules during the pre-freeze chilling and cryopreservation of NK cells in osmolyte solutions that limits the function of NK cells post-thaw. Cryopreservation of NK cells remains a challenge, largely due to intracellular damage incurred during freezing and thawing. Our findings indicate that cytolytic granules are particularly susceptible to destabilization under freezing stress. Damage to these granules can result in aberrant intracellular release of cytotoxic mediators, leading to immediate autolysis, delayed apoptotic pathways, and sustained impairment of NK cell function. The persistent reductions in post-thaw viability and cytotoxic capacity emphasize the critical role of granule integrity in successful NK cell preservation.

Across multiple DMSO-free cryopreservation formulations, immediate post-thaw recovery and viability consistently exceeded 80%, demonstrating effective short-term preservation. However, within 24 h of thawing, viability declined by 5%–10%, and the overall recovery decreased by 10%–20%, indicating the presence of delayed cell death mechanisms. Although NK cells resumed proliferation by 48 h post-thaw, expansion remained substantially attenuated compared with non-cryopreserved controls and did not reach comparable levels, even after 5 days in culture. Functional assessment revealed a reduction in NK cell-mediated cytotoxicity of at least 25% following thawing. While cytotoxic activity partially improved with rest in culture, it failed to return to pre-freeze levels, even after 5 days, underscoring the durability of cryo-induced functional deficits.

Chemical degranulation prior to freezing improved short-term recovery and proliferative capacity, yet it did not fully restore cytotoxic function. This finding suggests that granule removal alone is insufficient to prevent cryopreservation-associated dysfunction. Instead, our data support a multifactorial damage model involving mitochondrial stress, intracellular interactions with cryoprotective agents, and activation of delayed cell death pathways. DMSO-free cryopreservation solutions mitigate the well-documented toxicities associated with DMSO exposure and enable moderate functional recovery; however, full restoration of cytotoxic competence requires prolonged post-thaw culture, highlighting a disconnect between structural preservation and effector function.

Future studies will focus on refining cryopreservation strategies that minimize granule-associated injury while promoting durable functional recovery. In addition, future studies should validate these findings in primary human NK cells, although examining donor-to-donor variability in expansion, phenotype, cytotoxicity, and post-thaw recovery requires appropriately powered donor studies. Potential approaches include the optimization of pre-freeze degranulation or stimulation protocols, incorporation of protective additives targeting intracellular stress responses, and improved delivery or composition of cryoprotective agents to stabilize granules during freezing. Future studies should also evaluate whether cryopreservation-associated changes in NK cell function, granule organization, activation state, or cytoskeletal behavior alter the *in vivo* biodistribution and persistence after infusion, as freeze-thaw effects can affect the biodistribution and therapeutic properties of cellular therapeutics.^8^^,^^95^ Additionally, future studies should evaluate post-thaw secretion of cytokines, including IFN-γ and TNF-α, to determine whether cryopreservation alters NK cell effector function beyond target cell killing. Ultimately, cryopreservation methods that preserve both immediate viability and sustained NK cell effector function are important for advancing off-the-shelf NK cell therapy development, although future primary cell and *in vivo* studies are needed to establish therapeutic relevance.

## Material and methods

### Cell culture

NK-92 cells (ATCC CRL-2407) were cultured in MEM-alpha media (Gibco) supplemented with 12.5% fetal bovine serum (Gibco), 12.5% horse serum (Gibco), 400 U/mL human recombinant IL-2 (R&D Systems), and 0.1 mM 2-mercaptoethanol (Gibco). K562-GFP cells (ATCC CCL-243-GFP) were cultured in IMDM media (Gibco) supplemented with 10% fetal bovine serum (Gibco) and 0.5 μg/mL puromycin (Millipore). The cells were used at passages 3–11. Cell line verification was confirmed via short tandem repeat (STR) profiling (ATCC). All cell lines were tested for mycoplasma contamination, using a MycoAlert mycoplasma detection kit (Lonza). Aliquots of cells were stained with acridine orange and propidium iodide (AO/PI, Invitrogen) and enumerated with a Countess FL 3 (Invitrogen) to determine their viability and count.

### Cryoprotectant formulations

Combinations of sucrose (Millipore), glycerol (Humco), propylene glycol (Oakwood Chemical), ethylene glycol (Sigma), or isoleucine (Millipore), all with 99% purity or higher, were dissolved in Normosol-R, an isotonic carrier solution approved for infusion. DMSO (Fisher Scientific) was also prepared in Normosol-R or CryoStor 10 (BioLife Solutions).

### Cell cryopreservation

NK cells were harvested from culture, enumerated, centrifuged, and resuspended at twice (2×) the target concentration in Normosol-R for freezing. Final cell concentrations ranged from 5 to 10 million viable cells/mL. Formulations were added dropwise to the cells in a 1:1 volume ratio. The cells were incubated in the formulations for 60 min at room temperature prior to starting the controlled-rate freezing profile.

### Controlled-rate freezing and sample storage

A Kryo 560-16 (Planer) controlled rate freezer was used for cell freezing in 1.8 mL cryovials (Nunc).

1. Start temperature: 20°C
2. −10°C/min to 0°C
3. Hold at 0°C for 15 min
4. −1°C/min to −7°C
5. –35°C/min to −50°C
6. +10°C/min to −23°C
7. +2.2°C/min to −21°C
8. Cooling rate (CR) to −65°C
9. −10°C/min to −110°C.

After freezing, cryovials were transferred to storage in the vapor phase of liquid nitrogen.

### Cryovial thawing

Frozen cryovials were thawed in a 37°C water bath with gentle mixing until a sliver of ice remained (approximately 2 min 30 s). The cells were transferred to a conical tube and diluted by slow, dropwise addition of prewarmed media or isotonic solution. Cell enumeration was performed immediately after dilution to obtain post-thaw recovery and viability. Recovery is defined as the net number of viable cells post-thaw divided by the number of viable cells pre-freezing.

### DE algorithm

A DE algorithm was used to optimize a three-component osmolyte cryopreservation solution based on post-thaw viable cell recovery. The algorithm was implemented using the DE/rand/1/bin mutation strategy originally described by Storn and Price and adapted from prior cryopreservation optimization studies. Each candidate solution was represented as a vector containing concentrations of sucrose, glycerol, and isoleucine.

A 10% DMSO solution prepared in Normosol-R was used as a control solution. The composition parameter space was discretized into ten intervals between 0 and 333 mM sucrose, 0 and 10 wt/wt% glycerol, and 0 and 33 mM isoleucine.^44^^,^^59^^,^^60^^,^^96^

The compositions for Generation 0 were randomly generated within the defined parameter space. A population size of nine was used; so, nine unique formulations were tested per generation. NK-92 cells were cryopreserved in each formulation, thawed, and evaluated for post-thaw viable cell recovery. Recovery was normalized to the DMSO control and used as the objective function. Briefly, DE/rand/1/bin improves a population of candidate formulations by generating a trial formulation for each original formulation. Each trial formulation was created by combining information from randomly selected formulations within the same generation through mutation and crossover. The trial formulation was then experimentally tested. If the trial formulation produced equal or greater post-thaw recovery than the corresponding target formulation, it replaced thetarget formulation in the next generation; if not, the original formulation was retained. DE parameters included a scaling factor of 0.85 and a crossover rate of 1. Higher-performing candidate solutions were retained in the emergent population, and the algorithm was considered converged when the emergent population did not change between successive generations.

### Post-thaw proliferation

The thawed cells were diluted with or resuspended in media, transferred to culture flasks, and incubated at 37°C with 5% CO_2_. The cells were enumerated at least daily for up to 5 days post-thaw.

### Cytotoxicity assay

The Incucyte SX5 live-cell analysis system was used to determine NK cell-induced cytotoxicity against K562-GFP target cells. NK cells were co-cultured with target cells in a 96-well plate at different E:T ratios. Each well was imaged with the phase and green optical modules every hour for a duration of 24 h. Images were analyzed using the integrated software (Incucyte 2024B) to segment and enumerate the fluorescent target cells by top hat segmentation. NK cell-induced cytotoxicity after 24 h was reported as percent target cell reduction relative to target-only controls.

### Flow cytometry

The information for fluorophores, anti-human antibodies and manufacturers is shown in Table 1. The staining protocol is briefly described as follows: NK cells were stained with 7-aminoactinomycin D (AAD) for 10 min before fixation with 2% paraformaldehyde for 15 min. NK cells were then washed and resuspended in a staining buffer (PBS, 2% bovine serum albumin (BSA)). An antibody cocktail was added to the NK samples and incubated for 30 min at 4°C under a low-light environment. The samples were centrifuged for 8 min at 220 g, the supernatant was removed, and the cells were resuspended in buffer. Data were collected on a BD LSRFortessa X-20 with FACSDiva software. Cell gating included the range of forward and side scatter from single cells and dead cells.

**Table 1:** Antibody and fluorophore information

Antibody	Fluorophore	Manufacturer	Stain concentration
CD56	Alexa Fluor 488	BioLegend, 304611	5 μL/1 million cells
CD69	BV421	BioLegend, 310930	5 μL/1 million cells
CD107a	PE	BioLegend, 328607	5 μL/1 million cells
NKG2D	APC	BioLegend, 130212	5 μL/1 million cells
Viability	7-AAD	BD Pharmingen, 559925	5 μL/1 million cells

### Confocal Raman microscopy

A Peltier stage (Thermonamic Electronics) and a series 800 temperature controller (Alpha Omega Instruments) were used to cool or freeze NK cells on a microscope stage.^97^ The same cooling rates in controlled rate freezing can be obtained with this stage and cooling system. NK cell suspensions were prepared and frozen at a controlled rate. Ice nucleation was manually induced at −4°C by briefly touching a liquid nitrogen (LN_2_) chilled needle to the sample. The cells were cooled to −50°C where Raman data were collected. Raman spectroscopic measurements were made using the WITec Confocal Raman Microscope Alpha 300R with ultra-high-throughput spectrometer (UHTS) spectrometer and DV401 charge-coupled device (CCD) detector with 600/mm grating. A 532 nm Nd:YAG laser was used as the excitation source, and a 100× air objective (numerical aperture 0.90, Nikon) was used to focus the laser. Laser power at the objective was measured at 10 mW, using an optical power meter (Thorlabs). Spatial resolution (X-Y) of the microscope was measured by WITec at 305 nm in lateral direction and 790 nm axial (Z).

### Raman image analysis

Raman false-color images of different molecular targets were generated by integrating the Raman spectra, acquired at each pixel, across the characteristic wavenumber range (Table 2). The size of each pixel in the field of view was either 250 × 250 nm or 333 × 333 nm, and Raman spectra were obtained at each pixel, with a 0.2 s integration time. Raman false-color images of the components of interest were generated by discrete trapezoidal area integration (MATLAB) of various signal bands. Cell boundary identification was performed using a series of operations in MATLAB image analysis software, as previously described.^94^ Briefly, the cell boundary was identified using the amide I signal and masked to form an outline of the cell boundary. Ice boundaries were determined by creating a binary mask based on the mean value of the ice intensity at each pixel. If the signal was greater than the value of the mean, then it was determined to be ice. The remaining area that was not ice or cell was determined to be non-ice. Carotenoids and granules were determined using the same method as the cell and were overlaid onto the cell boundary and ice. Carotenoids and granules were imaged using a range of zero to the max. value of the signal in a distinct five-color legend and overlaid onto a binary image of the ice and non-ice areas, with the cell boundaries included as well. Spatial localization for target substances (amide I and cytochrome *c*) was evaluated by calculating Moran’s I by using GeoDa (Univariate Moran’s I) on the false-color images. Using a weighting matrix based on the eight nearest neighboring pixels, the spatial dispersion vs. autocorrelation range was between −1 and +1. A perfect correlation of signal locations and intensities corresponds to a Moran’s I of +1, a perfect dispersion of signals corresponds to −1, and a perfectly random distribution corresponds to 0.

**Table 2:** Characteristic Raman spectral peaks

Substance	Wavenumber range (cm^−1^)	Peak wavenumber (cm^−1^)	Assignment
DMSO	650–740	673	symmetric CS stretching^94^
Cytochrome *c*	1,117–1,137	1,127	heme vibration^63^
All CPAs (sucrose, glycerol, and isoleucine)	815–885	840	C–C stretching^44^
Carotenoid	1,140–1,170	1,155	C–C vibrations^61^
Carotenoid	1,502–1,535	1,525	conjugated C=C bonds^61^
Proteins and lipids	1,628–1,695	1,660	amide I C=C stretching^94^^,^^98^
Lipid	2,850–2,940	2,883	anti-symmetric C–H_2_^99^^,^^100^^,^^101^
Ice	3,087–3,162	3,125	OH stretching^94^

### Degranulation

NK cells were incubated with 2.5–50 ng/mL PMA and 50–1,340 nM ionomycin for 24 h in a 37°C incubator. Cell recovery after degranulation was determined by dividing the viable cell count at 24 h by the initial viable cell count. The percent degranulation was determined using flow cytometry, with degranulated cells determined by gating the CD3^−^/CD56^+^/CD107A^+^ population compared with CD3^−^/CD56^+^ population.

### Statistics

Statistical analyses were performed with RStudio (version 2024.04). Pairwise comparisons between two groups were performed using two-tailed one-way ANOVA, and post hoc pairwise comparisons were used for comparisons involving more than two groups, including Moran’s I analyses. Values less than 0.05 were considered significant. *p* values are shown as ∗ if *p* < 0.05, ∗∗ if *p* < 0.01, ∗∗∗ if *p* < 0.005, and ∗∗∗∗ if *p* < 0.001. Error bars indicate the standard deviation, unless indicated otherwise.

## Data and code availability

Data will be made available on reasonable request.

## Acknowledgments

This study was funded by the 10.13039/100000001National Science Foundation (grant number PFI-TT 2042111). Parts of this work were carried out in the Characterization Facility, 10.13039/100007249University of Minnesota, which receives partial support from the NSF through the MRSEC (award number DMR-2011401) and the NNCI (award number ECCS-2025124) programs. Part of this work was supported by the resources and staff at the University of Minnesota University Imaging Centers (UIC). SCR_020997.

## Author contributions

Conceptualization, A.J. and A.H.; methodology, A.J.; investigation, A.J., T.L., and A.S.M.; formal analysis, A.J. and T.L.; visualization, A.J.; writing – original draft, A.J.; writing – review & editing, T.L. and A.H.; supervision, A.H. All authors have contributed to the manuscript preparation and approved the submitted version.

## Declaration of interests

A.J. declares a conflict of interest as he holds equity in Evia Bio Inc., which is related to the subject of this research. However, the research was conducted independently through the University of Minnesota, and the company had no direct involvement in the study design, execution, or analysis. A.H. declares a conflict of interest as she holds equity in Evia Bio Inc. and has issued patents #10314302 and #20220240499 and an international patent application #PCT/US2020/029847 related to this work.
